# Osteopontin-integrin interaction as a novel molecular target for antibody-mediated immunotherapy in adult T-cell leukemia

**DOI:** 10.1186/s12977-015-0225-x

**Published:** 2015-11-24

**Authors:** Naoyoshi Maeda, Takashi Ohashi, Haorile Chagan-Yasutan, Toshio Hattori, Yayoi Takahashi, Hideo Harigae, Hiroo Hasegawa, Yasuaki Yamada, Masahiro Fujii, Katsumi Maenaka, Toshimitsu Uede

**Affiliations:** Division of Molecular Immunology, Institute for Genetic Medicine, Hokkaido University, Kita-15, Nishi-7, Kita-ku, Sapporo, 060-0815 Japan; Research Center for Infection-associated Cancer, Institute for Genetic Medicine, Hokkaido University, Kita-15, Nishi-7, Kita-ku, Sapporo, 060-0815 Japan; Division of Molecular Virology, Institute for Genetic Medicine, Hokkaido University, Kita-15, Nishi-7, Kita-ku, Sapporo, 060-0815 Japan; Laboratory of Disaster-Related Infectious Diseases, International Research Institute of Disaster Science, Tohoku University, 1-1 Seiryo-machi, Aoba-ku, Sendai, Miyagi 980-8574 Japan; Department of Pathology, Tohoku University Hospital, 1-1 Seiryo-machi, Aoba-ku, Sendai, Miyagi 980-8574 Japan; Department of Hematology and Rheumatology, Tohoku University Graduate School of Medicine, 1-1 Seiryo-machi, Aoba-ku, Sendai, Miyagi 980-8574 Japan; Department of Laboratory Medicine, Nagasaki University Hospital, 1-7-1 Sakamoto, Nagasaki, 852-8102 Japan; Division of Virology, Niigata University Graduate School of Medical and Dental Sciences, 1-757 Asahimachi-Dori, Niigata, Niigata 951-8510 Japan; Laboratory of Biomolecular Science, Faculty of Pharmaceutical Sciences, Hokkaido University, Kita-12, Nishi-6, Kita-ku, Sapporo, 060-0812 Japan; Center for Research and Education on Drug Discovery, Faculty of Pharmaceutical Sciences, Hokkaido University, Kita-12, Nishi-6, Kita-ku, Sapporo, 060-0812 Japan

**Keywords:** Adult T-cell leukemia, Osteopontin, Integrin, Cancer-associated fibroblasts, NOD/Shi-*scid*,*IL*-*2Rg*^*null*^ mouse, Monoclonal antibody

## Abstract

**Background:**

Adult T-cell leukemia (ATL) is a CD4^+^ T-cell neoplasm with a poor prognosis. A previous study has shown that there is a strong correlation between the secreted matricellular protein osteopontin (OPN) level and disease severity in ATL patients. Here, we investigated the role of OPN in ATL pathogenesis and the possible application of anti-OPN monoclonal antibody (mAb) for ATL immunotherapy in NOD/Shi-*scid*,*IL*-*2Rg*^*null*^ (NOG) mice.

**Results:**

Subcutaneous inoculation of ATL cell lines into NOG mice increased the plasma level of OPN, which significantly correlated with metastasis of the inoculated cells and survival time. Administration of an SVVYGLR motif-recognizing anti-OPN mAb resulted in inhibition not only of tumor growth but also of tumor invasion and metastasis. The number of fibroblast activating protein-positive fibroblasts was also reduced by this mAb. We then co-inoculated mouse embryonic fibroblasts (MEFs) isolated from wild-type (WT) or OPN knockout mice together with ATL-derived TL-OmI cells into the NOG mice. The mice co-inoculated with WT MEFs displayed a significant decrease in survival relative to those injected with TL-OmI cells alone and the absence of OPN in MEFs markedly improved the survival rate of TL-OmI-inoculated mice. In addition, tumor volume and metastasis were also reduced in the absence of OPN.

**Conclusion:**

We showed that the xenograft NOG mice model can be a useful system for assessment of the physiological role of OPN in ATL pathogenesis. Using this xenograft model, we found that fibroblast-derived OPN was involved in tumor growth and metastasis, and that this tumor growth and metastasis was significantly suppressed by administration of the anti-OPN mAbs. Our findings will lead to a novel mAb-mediated immunotherapeutic strategy targeting against the interaction of OPN with integrins on the tumor of ATL patients.

**Electronic supplementary material:**

The online version of this article (doi:10.1186/s12977-015-0225-x) contains supplementary material, which is available to authorized users.

## Background

Adult T-cell leukemia (ATL) is caused by the Human T-cell leukemia virus type 1 (HTLV-1) and is a highly aggressive CD4^+^ T-cell leukemia characterized by clonal integration of HTLV-1 in leukemic cells [1]. ATL is classified into four subtypes: acute, lymphoma, chronic, and smoldering [2]. As the prognosis of ATL patients remains extremely poor due to resistance to conventional chemotherapy regimens, introduction of new therapeutic agents is needed [3]. Indeed, many inhibitors and monoclonal antibodies targeting the tumor itself have been evaluated [4]. ATL cell invasion/metastasis is frequently observed in an early phase of disease progression, notably in the skin as well as the liver, lung and lymph nodes [5]. Thus, prevention of such invasion and metastasis could be another therapeutic strategy to prolong the survival time.

Cancer progression is known to be the result of complex crosstalk among different cell types in the primary tumor and its surrounding tissues [6]. The tumor microenvironment has a critical role in modulating and regulating the invasion and subsequent metastasis of many cancers [7]. It has been generally accepted that the extracellular matrix (ECM) formed by activated mesenchymal cells and secreted matricellular molecules in the tumor microenvironment play a critical role in tumorigenesis and tumor metastasis [8]. Interaction between E-selectin and sialyl Lewis^X^ [9, 10], between leukocyte function-associated antigen (LFA-1) and intracellular adhesion molecule (ICAM)-1 [11, 12], or between OX40 and gp34 [13], have been reported to be critical in ATL cell adhesion. Binding of the CC chemokine ligand (CCL)17 and CCL22 with the CC chemokine receptor (CCR)4, or of CCL19 and CCL21 with CCR7 is critical for tissue-specific metastasis [14].

Integrins, which are a large family of heterodimeric cell surface adhesion receptors, consist of one of 18 α and one of 8 β subunits [15]. It is well established that integrins are involved in tumor adhesion, invasion, and metastasis [16]. The α4β1 and α5β1 integrins are highly expressed in patients with lymphoma type ATL [17–19], and β7 integrins may be involved in gastrointestinal metastasis [20]. The matricellular molecule osteopontin (OPN) physiologically interacts with αvβ1, αvβ3, αvβ5, and α8β1 integrins via a classical cell-binding motif, the arginine-glycine-aspartic acid (RGD) sequence within the OPN molecule, or with α9β1 and α4β1 integrins via a serine-valine-valine-tyrosine-glycine-leucine-arginine (SVVYGLR) sequence within the OPN molecule [21]. It has been widely accepted that, upon this interaction, OPN regulates the development of various disorders including not only inflammatory and autoimmune diseases but also cancer development [22]. Thus, inhibition of the interaction of OPN with integrins could be an effective strategy for anti-tumor therapy [23, 24]. There is a strong correlation between the plasma OPN level and tumor burden, suggesting that plasma OPN could be a useful tumor marker in many cancer types [25]. We have reported a strong correlation between the OPN level and disease severity in ATL patients, which suggests that OPN is also involved in ATL development [26]. Moreover, CD68-positive macrophages and endothelial cells within tumor tissues express OPN, suggesting that stromal cell-derived OPN could be involved in the tumorigenesis of ATL. McAllister et al. have reported that secretion of OPN by instigating breast tumors is necessary for bone-marrow cell activation and the subsequent outgrowth of the distant indolent tumors in mice [27, 28]. On the other hand, we have recently proposed that stromal cell, especially cancer-associated fibroblast (CAF)-derived, secreted OPN is involved in tumor growth and metastasis in the breast tumor xenograft model [29].

In this study we investigated the physiological roles of OPN-integrin interaction on ATL pathogenesis in vitro and in vivo. In order to verify the function of mesenchymal stroma-derived OPN in ATL tumorigenesis in vivo, we used the NOD/Shi-*scid*,*IL*-*2Rg*^*null*^ (NOG) mouse [30] that has been widely utilized for analysis of ATL and other tumors [31]. Subcutaneous inoculation of ATL cell lines into NOG mice increased the plasma level of OPN, which significantly correlated with invasion of the inoculated cells and the survival time. Most importantly, treatment of tumor-bearing NOG mice with anti-OPN monoclonal antibodies (mAbs) resulted in inhibition not only of tumor growth but also of tumor invasion and metastasis. Our combined findings would lead to a novel OPN mAb-mediated immunotherapeutic strategy targeted towards the interaction of host stromal cell-derived OPN with integrins on tumor cells in ATL patients.

## Results

### OPN and integrin expression in tumor tissues and primary lymphocytes obtained from ATL patients

**Fig. 1:**
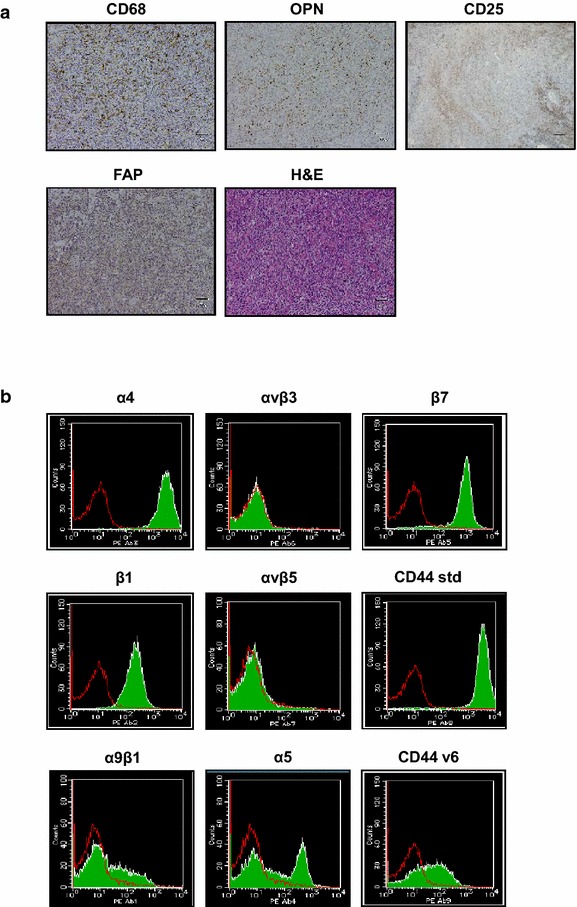
OPN expression in stromal cells of lymph nodes, and Integrin and CD44 expression in primary lymphocytes from ATL patients. **a** Human OPN, CD68, FAP, CD25, and H&E stained sections of ATL lymph nodes are shown. *Scale bars*, 50 μm. **b** PBMCs were isolated from patients with acute (n = 2) and chronic (n = 2) type ATL. The CD4^+^CD25^+^ population was gated, and the expression of integrins and of CD44 was evaluated using a FACS Calibur flow cytometer. *Green filled histograms* indicate cells stained with mAbs to the indicated integrins or CD44 variants. *Red open histograms* represent cells stained with isotype control IgGs. Data of a representative experiment from acute type ATL (primary ATL1 in Additional file 2: Table S1) are shown

We first assessed the expression of OPN in lymph nodes obtained from ATL patients (acute type and chronic type) using immunohistochemical staining. As previously reported by Changan-Yasutan et al., we detected human OPN expression in CD68-positive macrophages (Fig. 1a). Moreover, we also detected many fibroblast activation protein (FAP)-positive fibroblasts, called cancer-associated fibroblasts (CAFs), within the lymph nodes of ATL patients (Fig. 1a). The widespread OPN staining and the large number of FAP-positive fibroblasts suggests that the majority of CAFs express OPN. Based on the OPN staining pattern in comparison with CD4^+^CD25^+^ lymphocyte staining it seemed unlikely that the ATL cells themselves expressed OPN (Fig. 1a). Since we have previously reported an increase in plasma OPN levels in ATL patients [26], we next determined if this increase might be due to OPN secretion by the ATL cells themselves, by examination of the level of OPN secreted into the supernatant of ATL-derived cell lines and HTLV-I-transformed T cell lines. Unexpectedly but interestingly, we found that the cell lines secreted very little OPN into the supernatant (Additional file 1: Figure S1). We also tested if these cell lines expressed OPN receptors such as αvβ3, αvβ5, α4β1, and α9β1 integrins, or CD44std and CD44v6. FACS analysis indicated that each cell line expressed at least one of the OPN receptors on the cell surface. Further FACS analysis demonstrated that CD4^+^CD25^+^ T cells obtained from acute and chronic type ATL patients expressed α4β1, α5β1, and α9β1 integrins, and CD44std and CD44v6, while the expression of RGD motif-recognizing αvβ3 and αvβ5 integrins was extremely low or undetectable (Fig. 1b; Additional file 2: Table S1).

To address the involvement of OPN in the invasion process of ATL cells, we conducted an in vitro matrigel invasion assay. For this assay, we chose three cell lines with different integrin and OPN expression patterns; the in vitro HTLV-1 transformed T cell line SLB-1 (OPN^+^, αvβ3^+^, α4β1^−^, α9β1^+^, and CD44v6^+^), and two ATL-derived cell lines TL-OmI (OPN^−^, αvβ3^−^, α4β1^+^, α9β1^+^, and CD44v6^−^) and 43Tb(−) (OPN^−^, αvβ3^−^, α4β1^+^, α9β1^−^, and CD44v6^−^). Assay of their invasive ability indicated that the SLB-1 cells displayed the highest invasive ability of the three cell lines tested (Additional file 3: Figure S2). To evaluate the role of OPN in the invasion process, we treated the cell lines with an SVVYGLR motif-recognizing anti-OPN mAb; this anti-OPN antibody partially but significantly reduced the invasion of both SLB-1 and TL-OmI cells in vitro (Additional file 4: Figure S3). Anti-αvβ3 integrin and anti-β1 integrin mAbs were also used in the assays. It is noteworthy that anti-αvβ3 mAb exhibited inhibitory effect on the invasion of SLB-1 cells, while anti-β1 mAb reduced the invasion of both SLB-1 and TL-OmI cells (Additional file 4: Figures S3). In contrast to its effect on cell invasion, these mAbs did not inhibit ATL cell growth in vitro (Additional file 5: Figure S4).

### Inoculation of in vitro transformed or ATL-derived T cell lines into NOG mice

**Fig. 2:**
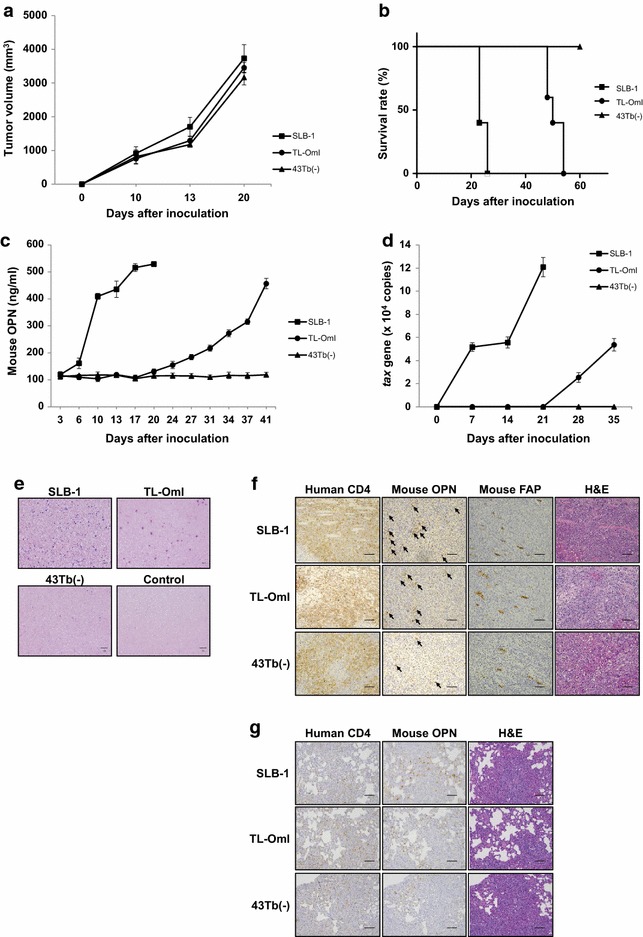
Host-derived OPN production correlates with pathogenicity in tumor cell-inoculated NOG mice. Six-week-old female NOG mice were subcutaneously inoculated with 5 × 10^7^ SLB-1 (n = 5), TL-OmI (n = 5), or 43Tb(−) (n = 5) cells. **a** The tumor size was measured every 3–4 days and is expressed in cubic millimeters. *Bars* indicate mean values ± SEM. **b** Survival rate was monitored based on death of the mice. **c** Blood was collected from the tail vein for measurement of the mouse OPN level in the plasma, and **d** for detection of metastatic cells as quantified by the number of *tax* genes assessed using qPCR. *Bars* indicate mean values ± SEM. For **a**–**d**, *filled squares*, SLB-1; *filled circles*, TL-OmI; *filled triangles*, 43Tb(−). **e** PBMCs were isolated on day 20 after the indicated cell inoculation and were stained with Giemsa. *Scale bars*, 50 μm. **f** Immunohistochemical staining of a subcutaneous tumor from an NOG mouse 20–50 days after the indicated cell inoculation. The sections were stained with Abs to the indicated proteins or with H&E. *Arrows* indicate the positive staining of mouse OPN. *Scale bars*, 100 μm. **g** Immunohistochemical staining of the lung from an NOG mouse 20–50 days after the indicated cell inoculation. The sections were stained with Abs to the indicated proteins or with H&E. *Scale bars*, 100 μm

To further evaluate the biological function of OPN in vivo, we subcutaneously inoculated the SLB-1, TL-OmI, and 43Tb(−) cell lines into NOG mice as described previously [32, 33]. The three cell lines inoculated into NOG mice produced visible tumors within 3 weeks (Fig. 2a). However, the SLB-1 tumor-bearing NOG mice had a poor prognosis, whereas the NOG mice inoculated with TL-OmI or 43Tb(−) survived longer than those inoculated with SLB-1 (Fig. 2b). It is noteworthy that the mouse OPN level in the plasma of SLB-1-inoculated NOG mice increased immediately after cell inoculation, while the increase in mouse OPN levels in the plasma of TL-OmI-inoculated NOG mice was only detectable at 24 days after inoculation. Mouse OPN levels were not significantly increased in 43Tb(−)-inoculated NOG mice up to 40 days after cell inoculation (Fig. 2c). Tumor-derived human OPN levels in plasma were negligible in comparison with the levels of host-derived murine OPN (data not shown). The data in Fig. 2c indicate that, similar to a previous study of human ATL patients [26], plasma OPN levels correlated well with poor prognosis in the xenografted NOG mice, strongly suggesting that this mouse model could be a useful tool for assessment of the physiological role of host-derived OPN in the pathogenesis of ATL. Very interestingly, the number of metastatic cells as judged by the appearance of ATL cells in the peripheral blood of NOG mice (assessed by assay of the HTLV-1 *tax* gene) correlated well with the plasma OPN levels (Fig. 2d). The level of ATL cells in the peripheral blood of NOG mice was higher when the mice were inoculated with SLB-1 cells than when they were inoculated with TL-OmI or 43Tb(−) cells as judged by Giemsa staining (Fig. 2e). Metastasis of SLB-1 cells, as judged by human CD4 staining of a subcutaneous tumor and of the lung, was also striking in the lung (Fig. 2f, g). As expected, while tumor cell-derived human OPN could not be detected in metastatic tissues (Additional file 6: Figure S5), host cell-derived mouse OPN was detected (Fig. 2f). We also detected the large number of FAP-positive fibroblasts in the primary SLB-1 tumors, suggesting that the majority of CAFs express OPN (Fig. 2f). In addition, OPN expression in the lung with metastatic SLB-1 cells was higher than that with metastatic TL-OmI or 43Tb(−) cells (Fig. 2g). These results indicated that host cell-derived OPN played an important role in tumor growth and metastasis.

### Treatment of tumor-bearing NOG mice with anti-OPN mAbs

**Fig. 3:**
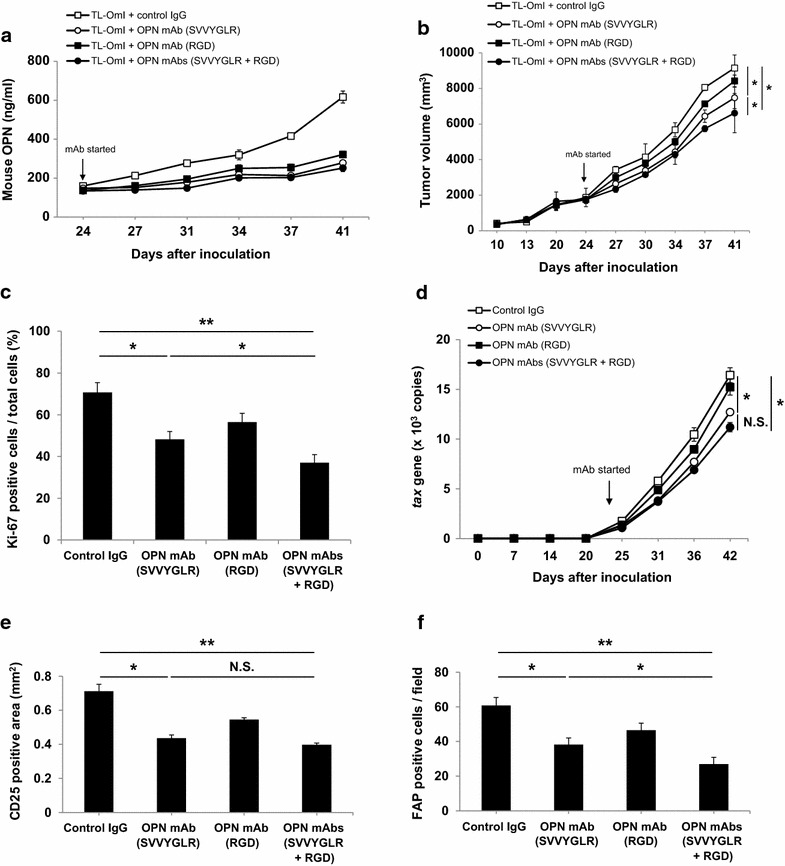
Anti-OPN mAbs suppressed the tumor growth and metastasis of ATL cells inoculated into NOG mice. Six-week-old female NOG mice were subcutaneously inoculated with 2 × 10^7^ TL-OmI cells. At 24 days after cell inoculation, the indicated anti-OPN mAbs (n = 5) or control IgG (n = 5) were intraperitoneally administered into the mice every 3–4 days. **a** Blood from the tail vein was collected for measurement of the mouse OPN level in the plasma. **b** The tumor size was measured every 3–4 days. **c** Immunohistochemical detection of tumor proliferation assessed by Ki-67 staining on day 40 after cell inoculation. **d** Blood from the tail vein was collected and metastatic cells were quantified by the number of *tax* genes assessed using qPCR. **e** Immunohistochemical detection of tumor metastasis by CD25 staining of liver tissue on day 40. **f** Immunohistochemical detection of CAFs by FAP staining of tumor tissues on day 40. For all of **a**–**f**, *bars* indicate mean values ± SEM. Statistically significant differences are shown as *P* values (*P < 0.05, **P < 0.01). *NS* no significant difference. For **a**, **b** and **d**, *open squares*, control IgG; *filled squares*, OPN RGD motif-recognizing mAb; *open circles*, OPN SVVYGLR motif-recognizing mAb; *filled circles*, OPN SVVYGLR motif-recognizing + RGD motif-recognizing mAbs

To further investigate the role of OPN in ATL tumorigenesis, we intraperitoneally administered two different anti-OPN mAbs into TL-OmI tumor-bearing NOG mice, an SVVYGLR motif- and an RGD motif-recognizing mAb. Administration of these anti-OPN mAbs, singly or in combination, starting at 24 days after tumor cell inoculation, significantly suppressed the level of mouse OPN in plasma (Fig. 3a). Under this experimental condition, the SVVYGLR motif-recognizing anti-OPN mAb significantly inhibited tumor growth (Fig. 3b). This inhibition correlated well with a reduction in Ki-67-positive cells in the primary tumors (Fig. 3c). It is of note that this anti-OPN mAb failed to inhibit ATL cell growth in vitro as shown in Additional file 5: Figure S4. The discrepancy between the in vivo and in vitro effect of this anti-OPN mAb led us to speculate that this anti-OPN mAb might indirectly inhibit tumor growth via suppression of stromal cell function in vivo. In addition to the inhibition of tumor growth in vivo, the SVVYGLR motif-recognizing anti-OPN mAb also suppressed the metastasis of inoculated ATL cells into peripheral blood (Fig. 3d) and metastasis to the liver (Fig. 3e) in vivo, suggesting that the SVVYGLR sequence in OPN is critical for regulating tumor growth and metastasis. We observed similar effects of the SVVYGLR motif-recognizing anti-OPN mAb on SLB-1-inoculated NOG mice (Additional file 7: Figure S6). Since OPN is produced by the host cells, as shown Fig. 2c, we examined the effect of this anti-OPN mAb on host cells in the tumor tissue. Since there were a large number of FAP-positive (CAF) cells in the tumor (Fig. 1a), we examined the effect of this antibody on the number of FAP-positive cells. We found that the number of FAP-positive CAFs in the primary tumor tissues was reduced by the SVVYGLR motif-recognizing anti-OPN mAb on day 40 after inoculation, indicating that OPN is required for the recruitment of FAP-positive fibroblasts to the tumor (Fig. 3f). On the other hand, the RGD motif-recognizing anti-OPN mAb was less effective than the SVVYGLR motif-recognizing anti-OPN mAb, emphasizing that targeting the SVVYGLR sequence rather than the RGD sequence in OPN is critical for controlling tumor development in this mouse model. However, interestingly, co-administration of the two anti-OPN mAbs exhibited cooperative effects on tumor growth (Fig. 3a–f). The combined data indicated that OPN regulated primary tumor growth by the recruitment of CAFs and by mediating tumor metastasis via its binding to α9β1 and/or α4β1 integrins.

**Fig. 4:**
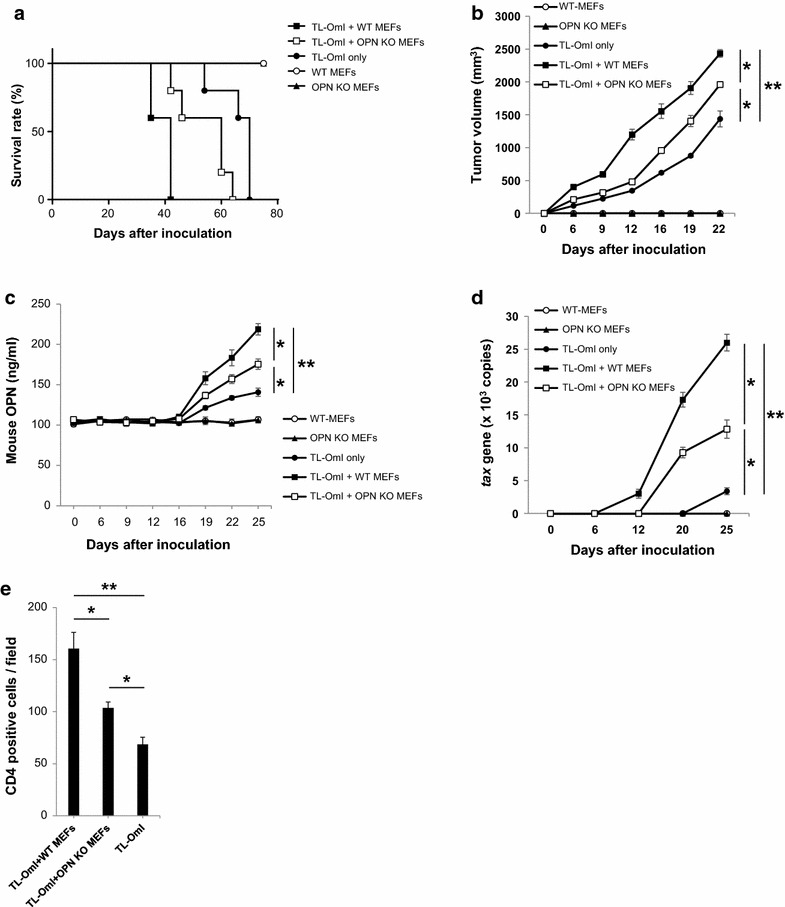
OPN produced by MEFs promotes the tumor growth and metastasis of ATL cells inoculated into NOG mice. Six-week-old female NOG mice were subcutaneously inoculated with TL-OmI cells alone (n = 5) or together with either WT MEFs (n = 5) or OPN KO MEFs (n = 5) (1 × 10^7^ cells per cell type). WT-MEFs (n = 3) or OPN KO MEFs (n = 3) only were also inoculated as negative controls. **a** Survival rate was monitored by death of the mice. **b** The tumor size was measured every 3–4 days. **c** Blood was collected from the tail vein for measurement of the mouse OPN level in the plasma and for **d** detection of metastatic cells as quantified by the number of *tax* genes assessed using qPCR. **e** Metastasis to the liver was evaluated by immunohistochemical staining of human CD4 on day 40. For all of **b**–**e**, *bars* indicate mean values ± SEM. Statistically significant differences are shown with *P* value (*P < 0.05, **P < 0.01). For **a**–**d**, *filled square*, TL-OmI + WT MEFs; *open square*, TL-OmI + OPN KO MEFs; *filled circle*, TL-OmI only; *open circle*, WT MEFs; *filled triangle*, OPN KO MEFs

### Co-inoculation of an ATL-derived cell line with mouse embryonic fibroblasts

To further investigate the biological role of CAF-derived OPN on ATL tumorigenesis and tumor metastasis in vivo, we subcutaneously inoculated mouse embryonic fibroblasts (MEFs) isolated from wild-type (WT) or OPN knockout (KO) mice [34] together with TL-OmI cells into the NOG mice. The mice co-inoculated with OPN KO MEFs displayed a significant increase in survival relative to those co-inoculated with WT MEFs (Fig. 4a). The absence of MEFs further improved the survival rate of TL-OmI-inoculated mice (Fig. 4a). Tumor growth was also evaluated by measuring the size of subcutaneous tumors. As shown in Fig. 4b, significantly lower tumor growth was observed in TL-OmI-injected mice co-inoculated with OPN-deficient MEFs compared with those co-inoculated with WT MEFs. Tumor growth was even lower in mice inoculated with TL-OmI alone. Inoculation of WT or OPN-deficient MEFs alone did not induced tumor formation at the inoculation sites (Fig. 4b). Although the plasma OPN level in TL-OmI/OPN KO MEF-inoculated mice was lower than that in TL-OmI/WT MEF-inoculated mice, it was still significantly higher than that in mice inoculated with TL-OmI alone, indicating that other factor(s) are involved in OPN production (Fig. 4c). Metastasis of inoculated cells correlated well with tumor growth (Fig. 4d). We further examined if the OPN derived from MEFs is involved in metastasis of the tumor cells. Metastasis to the liver was evaluated on day 40 after cell inoculation. Consistent with the data in Fig. 4d, the number of metastatic cells as assessed by immunohistochemical staining of CD4 in the liver was significantly increased in TL-OmI/WT MEF-inoculated mice compared with TL-OmI-inoculated mice, and the absence of OPN in the co-inoculated MEFs resulted in a lower number of tumor cells in the liver (Fig. 4e). We observed similar effects of the MEFs on tumor growth of 43Tb(−)-inoculated NOG mice (Additional file 8: Figure S7a). We detected metastatic cells in 43Tb(−)/MEF-inoculated mice, but the number of metastatic 43Tb(−) cells was lower than that in mice inoculated with SLB-1 alone or TL-OmI alone (Additional file 8: Figure S7b). Under this condition, the mouse OPN level in the plasma of 43Tb(−)/MEF-inoculated NOG mice was not increased up to 40 days after cell inoculation (data not shown), suggesting that the level of host-derived OPN in plasma is dependent on the number of metastatic cells. These combined data strongly suggested that the fibroblasts could be involved not only in tumor growth but also in tumor metastasis of ATL.

## Discussion

Cancer-related mortality is largely caused by tumor metastasis, therefore an understanding of the molecular mechanisms of metastasis is needed to develop a therapeutic anti-tumor strategy. ECM is a critical component of the microenvironment for tumor metastasis, and the matricellular molecule OPN plays an important role in this process. Based on our previous findings in a breast tumor model [29], the present study aimed to understand the biological function of OPN in the pathogenesis of ATL. Our present study provides new evidence that OPN could be involved in the tumorigenesis and tumor metastasis of ATL in a xenograft murine model. Furthermore, we designed and performed experiments using a novel therapeutic strategy that employed SVVYGLR motif- and RGD motif-recognizing anti-OPN mAbs in NOG mice. We demonstrate for the first time that interaction of host-derived OPN with tumor-derived integrins could be an attractive molecular target for mAb-mediated immunotherapy in ATL.

The relevance of elevated OPN production to human cancer pathogenesis has been shown [25]. However, the role of host-derived OPN in cancer pathogenesis has been unclear. In the case of ATL, we have previously reported that the prognosis was inversely correlated with the plasma OPN level, suggesting that OPN is also involved in ATL pathogenesis [26]. In this study we unexpectedly found ATL cell lines secreted very little OPN into the supernatant (Additional file 1: Figure S1). Among HTLV-I-infected cell lines, HTLV-I Tax protein is expressed in SLB-1 cells but not in TL-OmI cells and 43Tb(−) cells [35, 36]. Since the Tax protein activates OPN transcription mediated by a distal AP-1 site in the OPN promoter [37], it is likely that Tax expression and OPN production are correlated. We also found that NOG mice that were subcutaneously inoculated with ATL cell lines exhibited an increase in OPN levels in plasma (Fig. 2c). Very interestingly, the relationship between host-derived OPN production, survival time, and cell metastasis in the NOG mice was similar to that in ATL patients. The results of the cell invasion assay would be at least in part explained by the migration activity of each cell line in vitro (Additional file 3: Figure S2). It is of note that we found the three cell lines we used in this study had different integrin expression pattern (Additional file 2: Table S1). As shown in Additional file 4: Figure S3, the invasion process partially depends on the αvβ3 integrin and β1 integrin. Tuck et al. reported that more aggressive breast tumor cells preferentially used αvβ3 integrin but not αvβ5 or β1 integrins to respond to OPN [38], and we reported that α9β1 integrin is also involved in breast tumor metastasis [29]. In addition, OPN promotes tumor growth via αvβ3 integrin [39], or via α9β1 integrin [40] by distinct signaling pathways. These data suggest that the integrin expression pattern could be one of critical factors to determine the frequency of metastasis and tumor growth in ATL pathogenesis. The combined data suggest that the regulation of metastasis is critical for improving prognosis, and that host cell-derived OPN is involved in the metastasis observed in our xenograft model. Therefore, very importantly, this study has provided evidence that this NOG mouse model should be useful for evaluation of the biological involvement of not only OPN but also other associated genes in ATL pathogenesis in vivo. Besides OPN, other matricellular proteins that could interact with integrins such as tenascin-C [41], CCN-1 [42], or periostin [43] have not been reported in ATL patients, thus it will also be interesting to investigate a role of these molecules in patient samples and this NOG mouse model. On the other hand, we have previously reported an increase in plasma soluble CD44 (sCD44) levels in correlation with OPN in patient samples [26]. We found that HTLV-I-transformed cell lines and primary ATL cells express CD44 (Fig. 1b; Additional file 2: Table S1), which could be supported with an evidence that CD44 promoter is activated by HTLV-I Tax protein via noncanonical NF-κB pathway [44]. There is a strong correlation between the plasma sCD44 level and tumor development, suggesting that plasma sCD44 could be another useful tumor marker in many cancer types [45]. It is of great interest to investigate the relationship between OPN and sCD44 in ATL pathogenesis in our xenograft model.

The tumor microenvironment consists of a wide variety of non-tumor cell types such as fibroblasts, endothelial cells, and immune cells including macrophages [46]. Fibroblasts are the most abundant mesenchymal stroma cells (MSCs) within most carcinomas, and are widely regarded as CAFs that are definitely crucial for cancer development [47, 48]. Activated CAFs express molecules that could be useful as prognostic markers [49]. We previously found that OPN can be produced by activated fibroblasts in vivo, and we observed that these fibroblasts were induced to produce OPN either by direct interaction with tumor cells or by soluble factors derived from the tumor cells in vitro [29]. Indeed, in the present study we found that the number of FAP-positive cells was reduced by anti-OPN mAbs in primary tumor tissues, indicating that recruitment of CAFs are regulated via host-derived OPN (Fig. 3f). Moreover, tumor growth and metastasis were augmented by the addition of exogenous WT MEFs but not by OPN KO MEFs (Fig. 4), strongly suggesting the involvement of stromal OPN in ATL pathogenesis in vivo. With regard to the mechanisms of augmented OPN production, our preliminary data suggested that OPN production by fibroblasts was induced by direct interaction with ATL cells or with soluble factors derived from ATL cells. It has been reported that OPN production is induced by stimulation of a number of cell types with IFN-γ [50], IL-1 [51], TGF-β [52], or TNF-α [53]. However, we failed to detect the production of these cytokines by the ATL cells examined in this study, suggesting that other molecule(s) might be involved in ATL-associated OPN production. It is also possible that ATL cells could be induced to produce these cytokines by interacting with stromal cells in vivo. We are currently investigating how fibroblasts are induced to produce OPN in ATL pathogenesis. On the other hand, we have observed strong OPN expression in CD68-positive macrophages [26] as also shown in Fig. 1, suggesting that macrophages are involved in ATL pathogenesis through the production of OPN. Tumor-associated macrophages (TAMs) have been considered as critical stromal cells in the tumor microenvironment [54], and OPN is indeed up-regulated in TAMs [55, 56]. Moreover, an increased number of TAMs was strongly associated with shortened survival in patients with classical Hodgkin’s lymphoma and provides a new biomarker for risk stratification [57]. Although NOG mice have severe and multiple immune dysfunctions, phagocytic activity of macrophages is functionally retained [58]. In addition, several growth factors such as PDGF, EGF, and FGF produced by TAMs are able to activate fibroblasts [59]. Thus, the anti-OPN mAbs might reduce recruitment and activation of fibroblasts into primary tumor via suppressing OPN produced by TAMs. Therefore it remains to be investigated whether activated macrophages are involved in ATL pathogenesis. It will be interesting to evaluate the effects of macrophage-depleting reagents (e.g. dichloromethylene diphosphonate-containing liposomes) on ATL progression in our mouse model.

Reducing metastasis should be a major strategy to cure cancer and improve prognosis. Previous studies support the idea that OPN can act as a potential target for cancer therapy [60, 61]. In this study we investigated the effects of two different mAbs against OPN in an NOG mouse model of ATL and found that these two mAbs exerted distinct effects on the growth and metastasis of ATL cells. Since α9β1 and α4β1 integrins, the target of the SVVYGLR motif-recognizing mAb, are strongly expressed in TL-OmI cells but the αvβ3 integrin, which is the target of RGD motif-recognizing mAb, is not, it is likely that the distinct Ab effects observed are due to the expression level of these integrins. However, co-administration of the two mAbs exerted cooperative effects on tumor growth. OPN is known to be cleaved by cellular proteases, especially thrombin-cleaved N-half OPN exposes SVVYGLR-motif, which is recognized by α9β1 and α4β1 integrins, while full-length OPN is recognized by αvβ1, αvβ3, αvβ5, α5β1, and α8β1 integrins [21]. The RGD motif-recognizing mAb could reduce the full-length OPN (Fig. 3a) that results in reducing thrombin-cleaved N-half OPN, which is recognized by the SVVYGLR motif-recognizing mAb. It would be one of the possible mechanisms of the cooperative effects by co-administration on tumor growth. Further studies are required to understand the molecular mechanisms of the synergistic effects of these mAbs. However, this study has provided the first evidence that anti-OPN mAbs can reduce tumor growth and metastasis mainly by suppressing host cell-derived OPN production (Fig. 3). These mAbs might also reduce the invasion and metastasis of ATL cells via suppression of the tumor-derived OPN, as speculated based on the in vitro invasion assay (Additional file 4: Figure S3). On the other hand, with regard to the receptors for OPN, we detected strong expression of α9β1 integrin, as well as of α4β1, α5β1 and β7 integrins, while the expression of αvβ3 and αvβ5 integrins that interact with OPN via the RGD motif was much lower or undetectable on human primary ATL cells. We recently showed that the α9β1 integrin in tumors regulates not only breast tumor growth and metastasis but also OPN production from CAFs in breast tumor xenograft models [29]. In that study we first developed and used an anti-α9β1 integrin mAb that could inhibit the binding of the synthetic peptide, SVVYGLR. This anti-α9β1 integrin mAb suppressed tumor growth and metastasis in the breast tumor model. Since we detected the expression of α9β1 integrin in primary ATL cells (Fig. 1), it may be of value to evaluate the anti-tumor activity of this anti-α9β1 integrin mAb in the ATL xenograft model. In addition, CD44v6 could be another interesting target for ATL therapy, because we also detected the expression of CD44v6 in primary ATL cells (Fig. 1b), similar to other cancer types [62]. Binding of CD44v6 to OPN is dependent on β1 integrin but independent of RGD-motif to promote cell motility and chemotaxis [63], thus combination of β1 integrin and CD44v6 mAbs would be an attractive strategy on cancer treatment. Currently anti-CD44v6 mAb bivatuzumab conjugated with the maytansine derivative DM1 has been evaluated [64]. The combined data suggest that mAb-mediated immunotherapy targeting the molecules involved in invasion and metastasis would be a promising therapeutic approach for effective management of ATL. It is well known that skin metastasis of ATL tumors depends at least in part on CCR4 preference [65]. Indeed, the anti-CCR4 mAb mogamulizumab, which showed significant anti-tumor activity against ATL cells in the NOG mice via enhanced antibody-dependent cell-mediated cytotoxicity (ADCC) [66], has been developed and subsequently approved for the treatment of relapsed or refractory ATL in Japan [67]. Thus, understanding how chemokines and chemokine receptors are involved in ATL tumor metastasis should be important for planning an effective therapeutic strategy. Whereas CCR4 molecules are predominantly expressed in T cells, CCR5 molecules are expressed on CAFs and TAMs [14]. Interestingly, Mi et al. reported that OPN induced the production of the chemokine CCL5 from MSCs [68]. Moreover, they hypothesized that tumor-derived but not host-derived OPN could promote tumor progression via the transformation of MSCs into CAFs, and through increased MSC migration to metastatic sites such as lung and liver. In support of this observation, Wu et al. reported that a deficiency of the *CCL3* or *CCR5* genes strongly reduced the number of metastatic foci in the lung in a murine renal cell carcinoma model [69]. The role of the association of not only tumor-derived OPN but also of host-derived OPN with chemokines in ATL pathogenesis remains to be further investigated. An evaluation of chemokine or chemokine receptor induction by OPN is under way.

## Conclusions

In this study, we have shown that a xenograft NOG mice model can be a useful system to assess the physiological role of OPN in ATL pathogenesis. Using this xenograft assay, we found that stromal cell-derived but not tumor-derived OPN levels were increased during the course of tumor development. We noted that plasma OPN levels correlated well with poor prognosis and the number of metastatic cells. Furthermore, we found that fibroblast-derived OPN was involved in tumor growth and metastasis, which was significantly suppressed by the anti-OPN mAbs. Again, we emphasize that this study contributes to the understanding of physiological OPN involvement in ATL pathogenesis. Based on our new findings, we here propose the use of anti-OPN mAb as a novel therapeutic agent targeting this matricellular molecule in the host ECM for the treatment of ATL.

## Methods

### Animals

NOG mice [30] were obtained from the Central Institute for Experimental Animals (Kawasaki, Japan). Wild-type (WT) BALB/c mice were purchased from Japan SLC (Hamamatsu, Japan). OPN knockout (KO) mice were generated [34] and backcrossed at least 10 generations to BALB/c mice. All mice were maintained under specific-pathogen-free conditions in the Laboratory of Animal Experiments, Institute for Genetic Medicine, Hokkaido University.

### Cells

The HTLV-I-transformed T cell lines, C5/MJ, HUT-102, MT-2, MT-4, and SLB-1, and the ATL-derived cell lines, KK1, KOB, LM-Y1, ST1, TL-OmI, and 43Tb(−), were grown in RPMI-1640 medium supplemented with 10 % heat-inactivated fetal bovine serum (FBS), penicillin (100 units/ml), and streptomycin (100 μg/ml). Recombinant human interleukin (IL)-2 (0.5 nM) was added to the culture of KK1, KOB, and LM-Y1. Mouse embryonic fibroblasts (MEFs) were isolated from E13.5 embryos of WT BALB/c or OPN KO mice. The isolated cells were treated with trypsin-EDTA at 37 °C for 30 min, and were cultured in D-MEM supplemented with 10 % heat-inactivated FBS, penicillin (100 units/ml), and streptomycin (100 μg/ml).

### Anti-OPN mAbs

Both anti-OPN mAbs were obtained by immunizing mice with the synthetic peptide VDVPNGRGDSLAYGLR. This sequence is located in the internal sequence of murine OPN.

### Fluorescence-activated cell sorting (FACS) analysis

The expression of human CD4, CD8, αvβ3, αvβ5, α4β1, α5β1, α9β1, and β7 integrins as well as the standard and variant form of CD44 (CD44std and CD44v6, respectively) in human cell lines were examined by using a FACS Calibur flow cytometer (BD Biosciences). Primary antibodies were purchased as follows; CD4 (MT310; DAKO), CD8 (DK25; DAKO), αvβ3 integrin (LM609; Millipore), αvβ5 integrin (15F11; Millipore), α4 integrin (P1H4; Millipore), α5 integrin (IIA1; BD Pharmingen), α9β1 integrin (Y9A2; Millipore), β1 integrin (HUTS-4; Millipore), β1 integrin (P4C10; CHEMICON INTERNATIONAL), β7 integrin (473207; R&D Systems, Inc., USA), CD44 std (SFF-2; Bender MedSystems), CD44v3-10 (2C5; R&D Systems, Inc., USA), CD44v6 (2F10; R&D Systems, Inc., USA), and Negative control IgG1, IgG2a, and IgG2b (Millipore). Primary ATL lymphocytes from patients with acute and chronic ATL were isolated and analyzed at Nagasaki University Hospital. This study was approved by the Ethics Committees of Nagasaki University Hospital. In the CD4^+^CD25^+^ fraction, the expression of αvβ3, αvβ5, α4β1, α5β1, α9β1, and β7 integrins, as well as of CD44std and CD44v6 was analyzed using flow cytometry. Background-corrected mean fluorescence intensity (MFI) was determined for each cell type.

### Enzyme-linked immunosorbent assay (ELISA)

Blood was collected from the mouse tail with heparin, and the plasma was isolated to measure the amount of mouse OPN. The level of mouse OPN in mouse plasma and of human OPN in the supernatant of human cell lines was measured using a Mouse or Human OPN ELISA kit (R&D Systems, Inc., USA), respectively.

### In vitro cell invasion assay

This assay was performed using a BD BioCoat Matrigel Invasion Chamber (BD Biosciences) according to the protocol provided by the manufacturer. Briefly, the lower wells were filled with RPMI-1640 medium without supplements. Cells (SLB-1, TL-OmI, and 43Tb(−)) were seeded at a density of 1 × 10^5^ cells in serum-free RPMI-1640 medium in the invasion chamber containing the matrigel membrane and were allowed to settle for 3 h at 37 **°**C. RPMI-1640 medium supplemented with 10 % heat-inactivated FBS was added to the lower compartment of the invasion chamber. The chambers were incubated in the presence of 50 μg/ml of anti-OPN mAb, anti-αvβ3 integrin mAb, anti-β1 integrin mAb or control IgG for 8–24 h at 37 **°**C. The invading cells appeared at the lower surface of the membrane. The upper surface of the membrane was scrubbed and the absence of cells at the upper surface was confirmed. After the cells were fixed with methyl alcohol and stained with Giemsa, the membrane was covered with immersion oil and a cover slip, and the cells were randomly counted under a microscope.

### In vitro cell proliferation assay

Cells (SLB-1 and TL-OmI) were seeded at a density of 1 × 10^5^ cells in the presence of 50 μg/ml of anti-OPN mAb, anti-αvβ3 integrin mAb, anti-β1 integrin mAb or control IgG for 72 h at 37 °C. The cells were further incubated with the Cell Proliferation Reagent WST-1 (Roche) for 2 h at 37 °C. The absorbance of the samples was measured at 450 and 550 nm (reference wave length) using a microplate reader.

### Quantitative PCR analysis of the HTLV-I *tax* gene

Total genomic DNA was extracted from PBMCs derived from NOG mice using the DNeasy Blood & Tissue Kit (QIAGEN) according to the protocol provided by the manufacturer. Quantitative PCR (qPCR) was performed using the LightCycler FastStart DNA Master^PLUS^ Hybridization Probes (Roche Applied Science, Germany). The PCR condition was as follows; 40 cycles of 94 °C for 30 s., 60 °C for 10 s., and 72 °C for 15 s. The primer sequences were as follows: forward primer, 5′-CCCGAAGACTGTTTGCCCA-3′, and reverse primer, 5′-GGAAATCATAGGCGTGCCATC-3′. Hybridization probe sequences were as follows: the fluorescein isothiocyanate (FITC)-labeled probe was 5′-ACGGCCTCCTTCCGTTCC-3′, and the LC Red 640-labeled probe was 5′-TCAACCCTCACCACTCCAGGCCTTATTTGGA-3′. To construct a standard curve, a *tax* DNA plasmid was serially 10-fold-diluted from 2 × 10^4^ to 2 × 10^1^ copies/μl.

### Histological and cytological staining

Blood smear slides were fixed with methyl alcohol and dried for Giemsa staining. Lung, liver, or tumor was excised from NOG mice inoculated with or without cell lines. Part of the excised organs were fixed with dry-ice acetone, embedded in Tissue-Tek OCT compound (Sakura Finetechnical Co. Ltd., Tokyo, Japan), and stored at −80 °C until use. Frozen sections of the organs were prepared using a Cryostat and were fixed in acetone at room temperature for 20 min. The rest of the excised organs were fixed with 4 % paraformaldehyde for H&E staining. These organs were also stained with antibodies against human OPN (O-17, IBL, Gunma, Japan), human CD4 (MT310, DAKO), human IL-2Rα (CD25) (R&D Systems, Inc., Minneapolis), mouse OPN (O-17, IBL, Gunma, Japan), human Ki-67 (Abcam), and mouse fibroblast activation protein (FAP) (rabbit polyclonal, Abcam). Tissue samples from patients with ATL were analyzed at Tohoku University Hospital. This study was approved by the Ethics Committees of Tohoku University Hospital. The tissues were also stained with H&E, human OPN (MPIIIB10_1_, DSHB), human FAP (rabbit polyclonal, Abcam), human CD25 (4C9, Roche), and a macrophage marker CD68 (PG-M1, DAKO) as described above.

### Tumor xenograft model

SLB-1, TL-OmI, and 43Tb(−) cells were washed twice with serum-free RPMI-1640 medium. These cells were re-suspended in serum-free RPMI-1640 medium and inoculated subcutaneously into the NOG mice at a density of 1–5 × 10^7^ cells per mouse. For therapeutic experiments, 400 μg of anti-OPN (SVVYGLR) mAb, 400 μg of anti-OPN (RGD) mAb, 200 μg of each anti-OPN (SVVYGLR) mAb and anti-OPN (RGD) mAb for co-administration, or 400 μg of isotype-matched control IgG were intraperitoneally injected into NOG mice twice a week from day 24 (TL-OmI) or day 3 (SLB-1) after cell inoculation. For co-inoculation experiments, fibroblasts from WT BALB/c or OPN KO mice were subcutaneously inoculated with or without TL-OmI cells or 43Tb(−) cells at a ratio of 1:1. The tumor size was measured and determined using the formula (L × W^2^)/2 (L; length, W; width). Statistically significant differences between mAb- and control IgG-treated mice were calculated using Student’s *t*-test and are indicated as *P* values. Differences of *P* <0.05 were considered statistically significant. All experiments were approved and performed in accordance with the guidelines of the Committee of Ethics on Animal Experiments in Hokkaido University.

## Additional files

10.1186/s12977-015-0225-x OPN secretion into the supernatant by human T-cell lines. The indicated human T cell lines (1 × 10^6^ cells per line) were cultured for 24 h at 37 °C. The supernatant was then harvested for measurement of human osteopontin (OPN) using ELISA kits. *Bars* indicate mean values ± SEM. Data are representative of three independent experiments.
10.1186/s12977-015-0225-x Integrins and CD44 expression on T cell lines and primary cells associated with ATL.
10.1186/s12977-015-0225-x Invasion of HTLV-I-infected or ATL-derived T cell lines in vitro. The cells (SLB-1, TL-OmI, or 43Tb(−)) were seeded at a density of 1 × 10^5^ cells in the upper part of a matrigel transwell, in RPMI-1640 supplemented with 10 % heat-inactivated FBS. The cells were then placed on top of a lower well, which was filled with RPMI-1640 without supplements. Cells that had migrated on or through the matrigel were fixed with methyl alcohol for H&E staining and were counted at 24 h after seeding. *Bars* indicate mean values ± SEM. Data are representative of three independent experiments.
10.1186/s12977-015-0225-x Inhibitory effects of an anti-OPN Ab on invasion of HTLV-I-infected or ATL-derived T cell lines in vitro. The cells (SLB-1 and TL-OmI) were seeded at a density of 1 × 10^5^ cells in the upper part of a matrigel transwell and were then placed on top of the lower wells. Anti-OPN mAb (SVVYGLR motif-recognizing), anti-αvβ3 mAb, anti-β1 mAb or control IgG were added at a concentration of 50 μg/ml, and the cells were cultured for 8–24 h at 37 °C. Cells that had migrated on or through the matrigel were fixed with methyl alcohol for Giemsa staining and were counted at 24 h after seeding. Scale bars, 100 μm. *Bars* indicate mean values ± SEM. Statistically significant differences are shown as *P* value (**P* < 0.05, ***P* < 0.01). Data are representative of three independent experiments.
10.1186/s12977-015-0225-x Effects of the SVVYGLR motif-recognizing anti-OPN Ab on the proliferation of HTLV-I-infected or ATL-derived T cell lines in vitro. The cells (SLB-1, TL-OmI) were seeded at a density of 1 × 10^5^ cells in the presence of 50 μg/ml of an anti-OPN mAb (SVVYGLR motif-recognizing), anti-αvβ3 mAb, anti-β1 mAb or control IgG in a 96-well plate, and were cultured for 72 h at 37 °C. The cells were then further incubated with the Cell Proliferation Reagent WST-1 (Roche) for 2 h at 37 °C. The absorbance of the samples was measured at 450 and 550 nm (reference wave length) using a microplate reader. *Bars* indicate mean values ± SEM. *N.S.*; no significant difference. Data are representative of three independent experiments.
10.1186/s12977-015-0225-x Immunohistological staining of a subcutaneous tumor and of the lung from an NOG mouse. Immunohistochemical staining of a subcutaneous tumor and of the lung from an NOG mouse 20 days after subcutaneous inoculation of SLB-1 cells. The sections were stained with Ab to the human OPN.
10.1186/s12977-015-0225-x Anti-OPN mAb suppressed the tumor growth and metastasis of SLB-1 cells inoculated into NOG mice. Six-week-old female NOG mice were subcutaneously inoculated with 2 × 10^7^ SLB-1 cells. At 3 days after cell inoculation, the indicated anti-OPN mAbs (n = 3) or control IgG (n = 3) were intraperitoneally administered into the mice every 3–4 days. (A) Blood from the tail vein was collected for measurement of the mouse OPN level in the plasma. (B) The tumor size was measured every 3–4 days. (C) Blood from the tail vein was collected and metastatic cells were quantified by the number of *tax* genes assessed using qPCR. (D) Immunohistochemical detection of tumor metastasis by CD4 staining of lung tissue on day 20. For all of (A) to (D), *bars* indicate mean values ± SEM. Statistically significant differences are shown as *P* values (*, *P* < 0.05). For (A)–(C), open squares, control IgG; open circles, OPN SVVYGLR motif-recognizing mAb.
10.1186/s12977-015-0225-x OPN produced by MEFs promotes the tumor growth and metastasis of 43Tb(−) cells inoculated into NOG mice. Six-week-old female NOG mice were subcutaneously inoculated with 43Tb(−) cells alone (n = 3) or together with either WT MEFs (n = 3) or OPN KO MEFs (n = 3) (1 × 10^7^ cells per cell type). WT-MEFs (n = 1) or OPN KO MEFs (n = 1) only were also inoculated as negative controls. (A) The tumor size was measured every 3–4 days. (B) Blood was collected from the tail vein for detection of metastatic cells as quantified by the number of *tax* genes assessed using qPCR. For (A) and (B), *bars* indicate mean values ± SEM. Statistically significant differences are shown with *P* value (**P* < 0.05, ***P* < 0.01). For (A) and (B), filled square, 43Tb(−) + WT MEFs; open square, 43Tb(−) + OPN KO MEFs; filled circle, 43Tb(−) only; open circle, WT MEFs; filled triangle, OPN KO MEFs.

## Abbreviations

ADCC: antibody-dependent cell-mediated cytotoxicity
ATL: adult T-cell leukemia
CAF: cancer-associated fibroblast
CCL: CC chemokine ligand
CCR: CC chemokine receptor
CD44std: CD44 standard
CD44v6: CD44 variant6
ECM: extracellular matrix
EGF: epidermal growth factor
ELISA: enzyme-linked immunosorbent assay
FACS: fluorescence-activated cell sorting
FAP: fibroblast activation protein
FGF: fibroblast growth factor
FITC: fluorescein isothiocyanate
H&E: hematoxylin and eosin
HTLV-I: human T-cell leukemia virus type I
ICAM: intracellular adhesion molecule
IFN: interferon
IL: interleukin
KO: knockout
LFA: leukocyte function-associated antigen
mAb: monoclonal antibody
MEF: mouse embryonic fibroblast
MFI: mean fluorescence intensity
MSC: mesenchymal stromal cell
NOG: NOD/Shi-*scid*,*IL*-*2Rg*^*null*^
OPN: osteopontin
PDGF: platelet-derived growth factor
qPCR: quantitative polymerase chain reaction
TAM: tumor-associated macrophage
TGF: transforming growth factor
TNF: tumor necrosis factor
WT: wild-type
