# Supplementary figures and images for: Osteopontin-integrin interaction as a novel molecular target for antibody-mediated immunotherapy in adult T-cell leukemia
Source: Retrovirology. 2015 Nov 24;12:99. doi: 10.1186/s12977-015-0225-x (PMC4657376; doi:10.1186/s12977-015-0225-x)

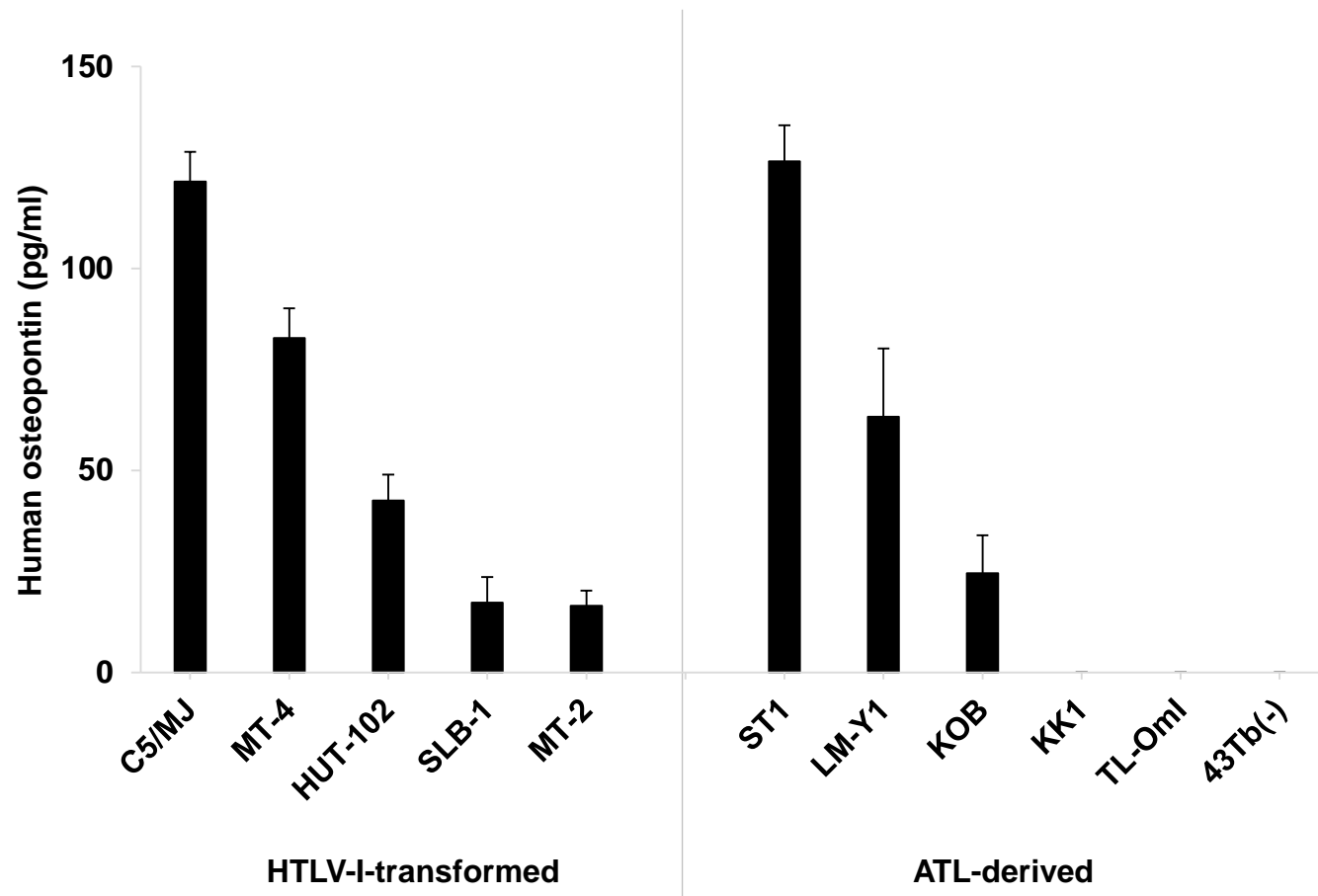

**Supplementary Figure S1**

Supplement: Supplementary file 1 — 10.1186/s12977-015-0225-x OPN secretion into the supernatant by human T-cell lines. The indicated human T cell lines (1 × 10^6^ cells per line) were cultured for 24 h at 37 °C. The supernatant was then harvested for measurement of human osteopontin (OPN) using ELISA kits. Bars indicate mean values ± SEM. Data are representative of three independent experiments. [file 12977_2015_225_MOESM1_ESM.pdf]

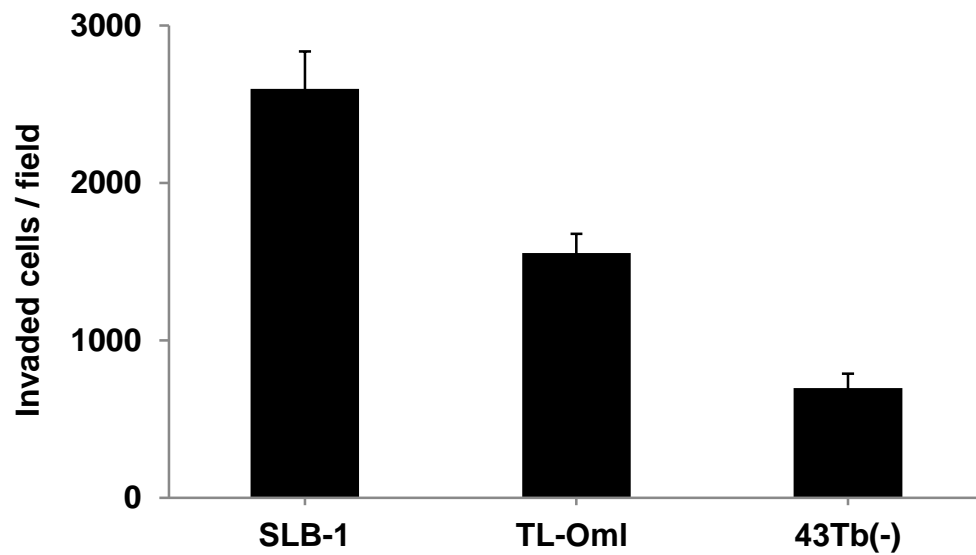

**Supplementary Figure S2**

Supplement: Supplementary file 3 — 10.1186/s12977-015-0225-x Invasion of HTLV-I-infected or ATL-derived T cell lines in vitro. The cells (SLB-1, TL-OmI, or 43Tb(−)) were seeded at a density of 1 × 10^5^ cells in the upper part of a matrigel transwell, in RPMI-1640 supplemented with 10 % heat-inactivated FBS. The cells were then placed on top of a lower well, which was filled with RPMI-1640 without supplements. Cells that had migrated on or through the matrigel were fixed with methyl alcohol for H&E staining and were counted at 24 h after seeding. Bars indicate mean values ± SEM. Data are representative of three independent experiments. [file 12977_2015_225_MOESM3_ESM.pdf]

**Figure S3**

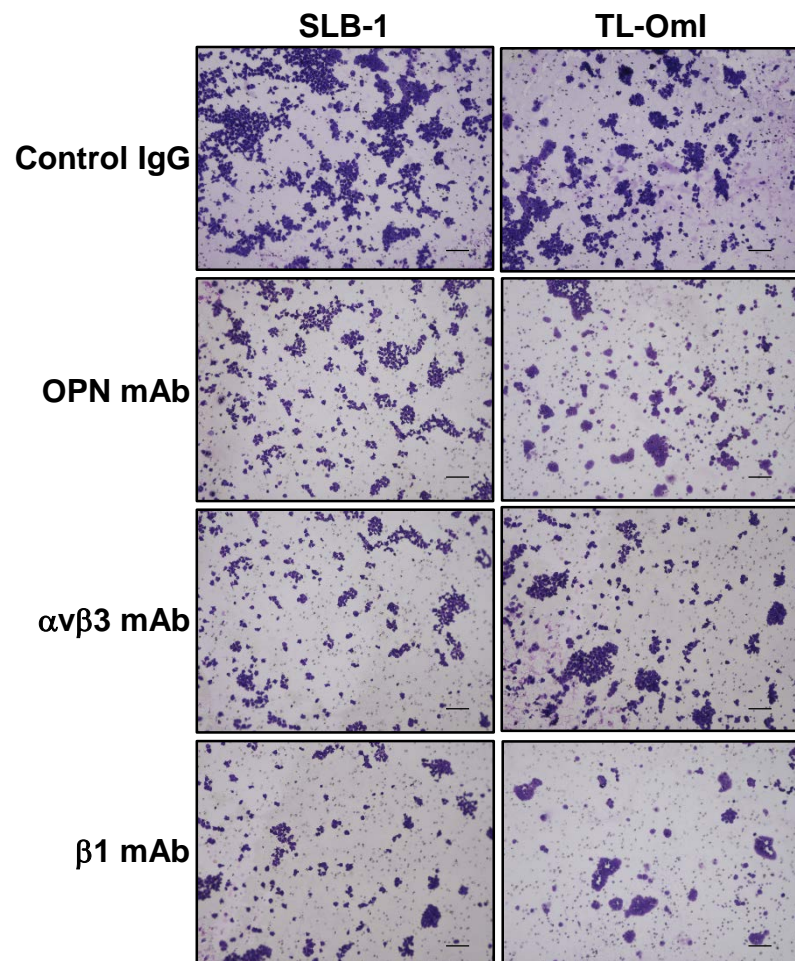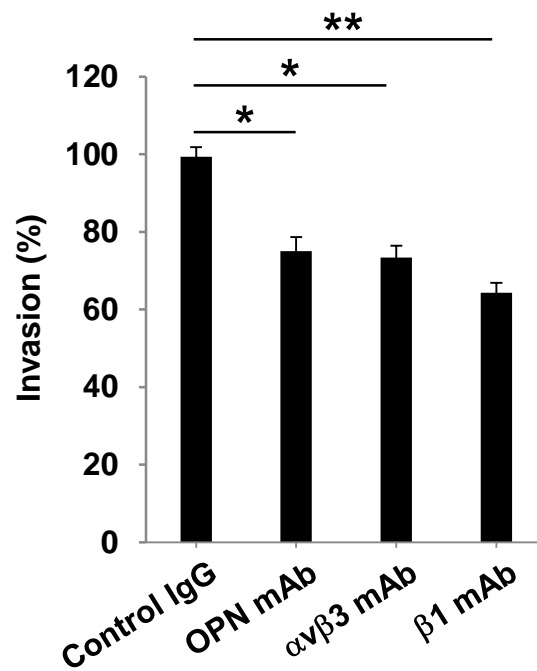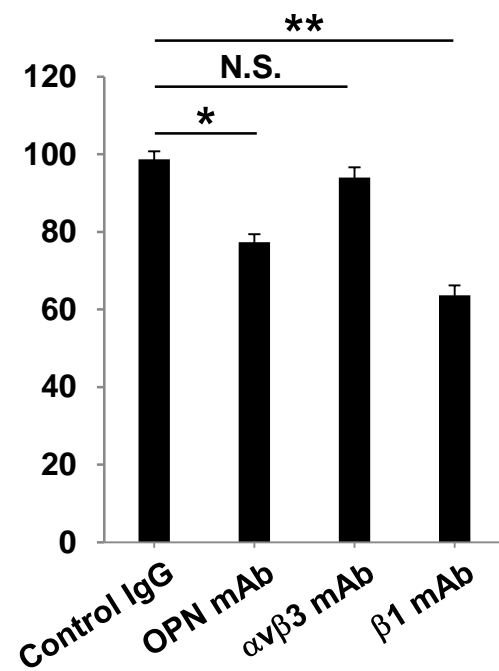

Supplement: Supplementary file 4 — 10.1186/s12977-015-0225-x Inhibitory effects of an anti-OPN Ab on invasion of HTLV-I-infected or ATL-derived T cell lines in vitro. The cells (SLB-1 and TL-OmI) were seeded at a density of 1 × 10^5^ cells in the upper part of a matrigel transwell and were then placed on top of the lower wells. Anti-OPN mAb (SVVYGLR motif-recognizing), anti-αvβ3 mAb, anti-β1 mAb or control IgG were added at a concentration of 50 μg/ml, and the cells were cultured for 8–24 h at 37 °C. Cells that had migrated on or through the matrigel were fixed with methyl alcohol for Giemsa staining and were counted at 24 h after seeding. Scale bars, 100 μm. Bars indicate mean values ± SEM. Statistically significant differences are shown as P value (*P < 0.05, **P < 0.01). Data are representative of three independent experiments. [file 12977_2015_225_MOESM4_ESM.pdf]

**Figure S4**

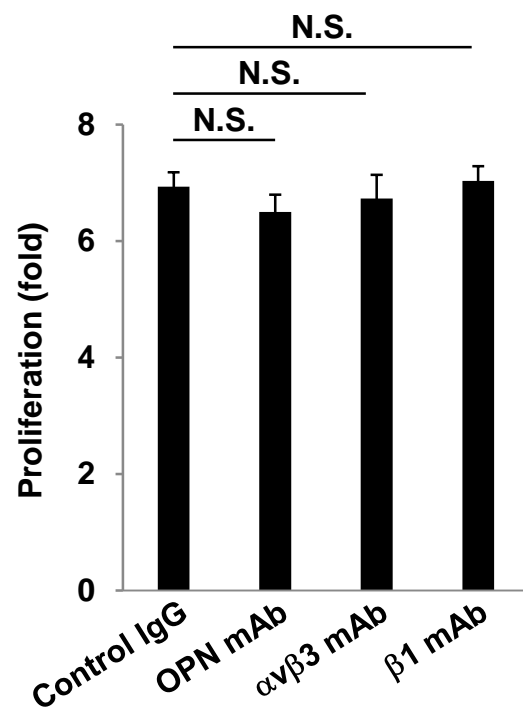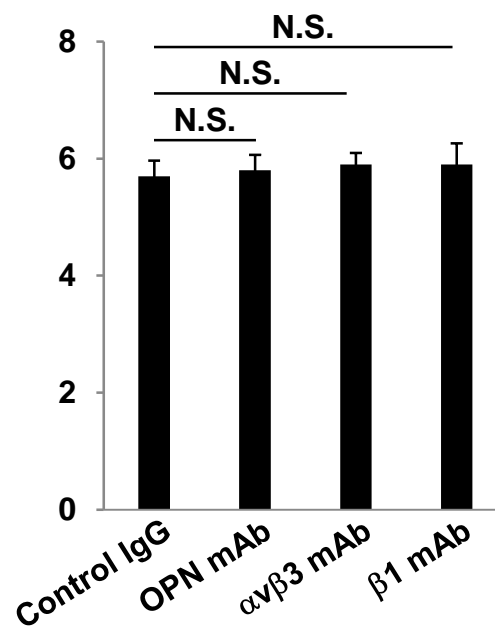

Supplement: Supplementary file 5 — 10.1186/s12977-015-0225-x Effects of the SVVYGLR motif-recognizing anti-OPN Ab on the proliferation of HTLV-I-infected or ATL-derived T cell lines in vitro. The cells (SLB-1, TL-OmI) were seeded at a density of 1 × 10^5^ cells in the presence of 50 μg/ml of an anti-OPN mAb (SVVYGLR motif-recognizing), anti-αvβ3 mAb, anti-β1 mAb or control IgG in a 96-well plate, and were cultured for 72 h at 37 °C. The cells were then further incubated with the Cell Proliferation Reagent WST-1 (Roche) for 2 h at 37 °C. The absorbance of the samples was measured at 450 and 550 nm (reference wave length) using a microplate reader. Bars indicate mean values ± SEM. N.S.; no significant difference. Data are representative of three independent experiments. [file 12977_2015_225_MOESM5_ESM.pdf]

**Figure S5**

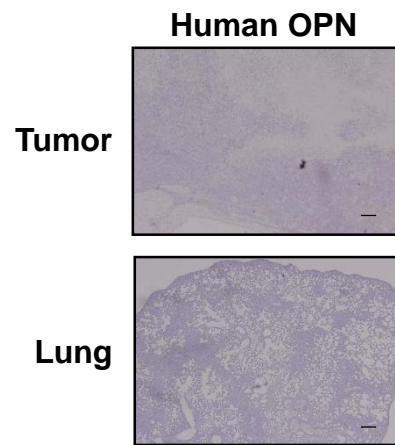

Supplement: Supplementary file 6 — 10.1186/s12977-015-0225-x Immunohistological staining of a subcutaneous tumor and of the lung from an NOG mouse. Immunohistochemical staining of a subcutaneous tumor and of the lung from an NOG mouse 20 days after subcutaneous inoculation of SLB-1 cells. The sections were stained with Ab to the human OPN. [file 12977_2015_225_MOESM6_ESM.pdf]

# Figure S6

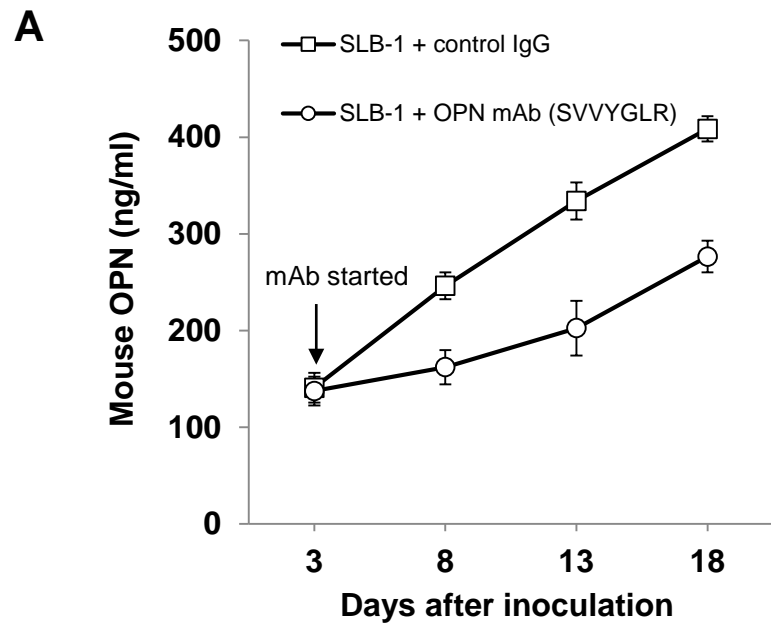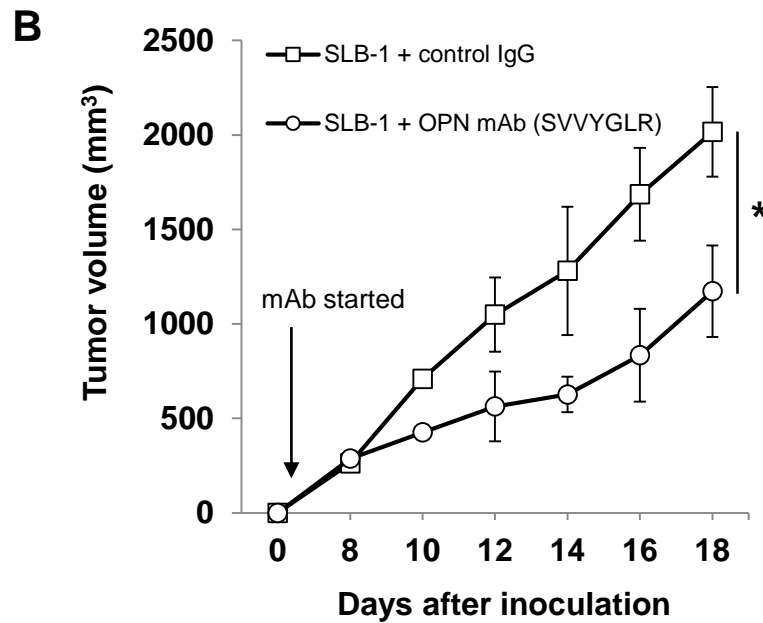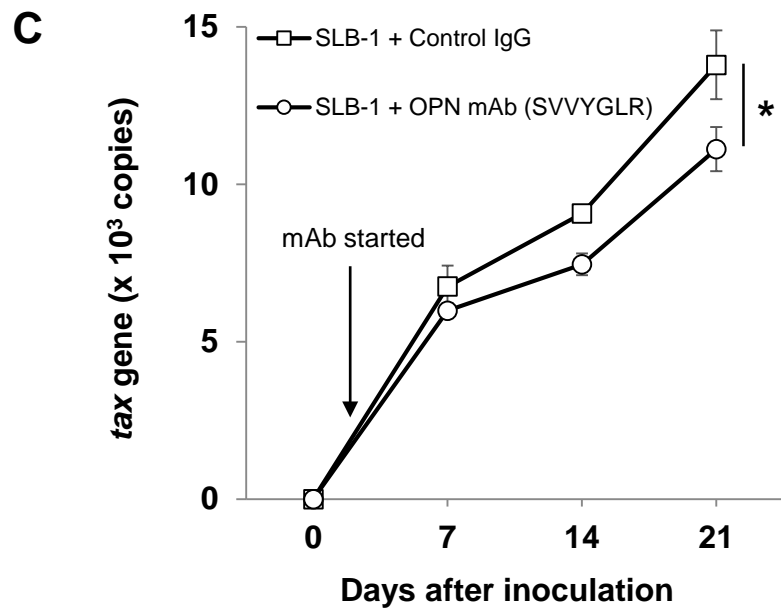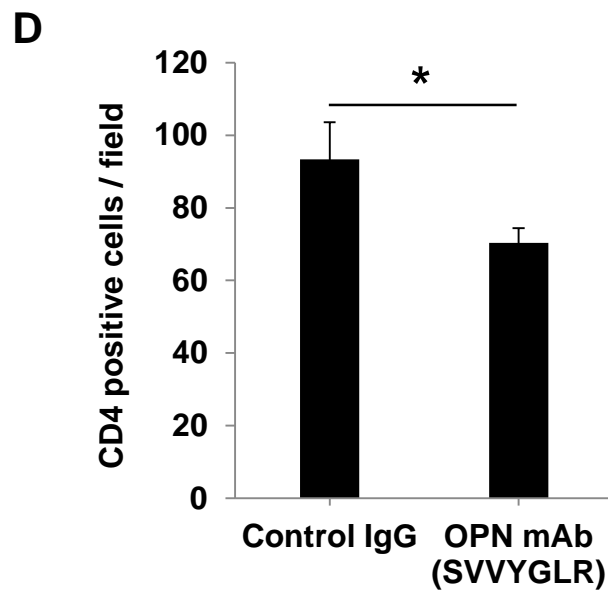

Supplement: Supplementary file 7 — 10.1186/s12977-015-0225-x Anti-OPN mAb suppressed the tumor growth and metastasis of SLB-1 cells inoculated into NOG mice. Six-week-old female NOG mice were subcutaneously inoculated with 2 × 10^7^ SLB-1 cells. At 3 days after cell inoculation, the indicated anti-OPN mAbs (n = 3) or control IgG (n = 3) were intraperitoneally administered into the mice every 3–4 days. (A) Blood from the tail vein was collected for measurement of the mouse OPN level in the plasma. (B) The tumor size was measured every 3–4 days. (C) Blood from the tail vein was collected and metastatic cells were quantified by the number of tax genes assessed using qPCR. (D) Immunohistochemical detection of tumor metastasis by CD4 staining of lung tissue on day 20. For all of (A) to (D), bars indicate mean values ± SEM. Statistically significant differences are shown as P values (*, P < 0.05). For (A)–(C), open squares, control IgG; open circles, OPN SVVYGLR motif-recognizing mAb. [file 12977_2015_225_MOESM7_ESM.pdf]

**Figure S7**

**A**

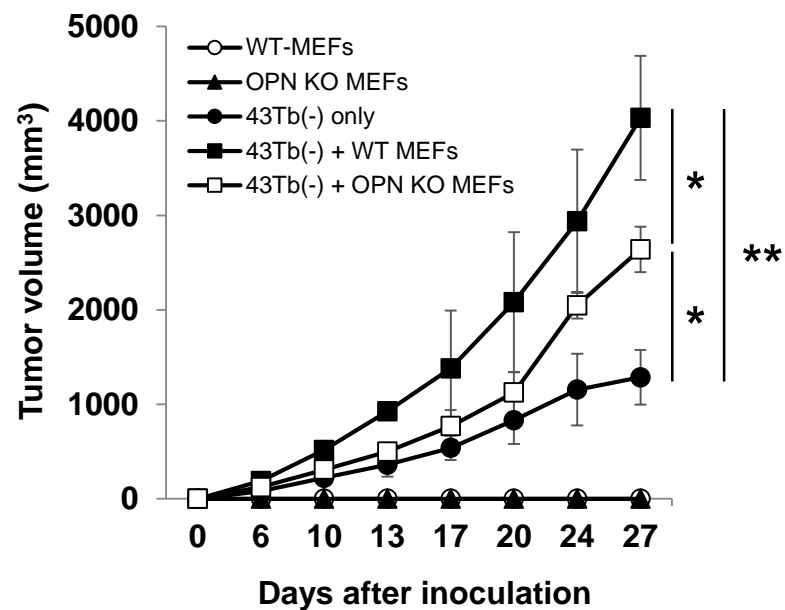

**B**

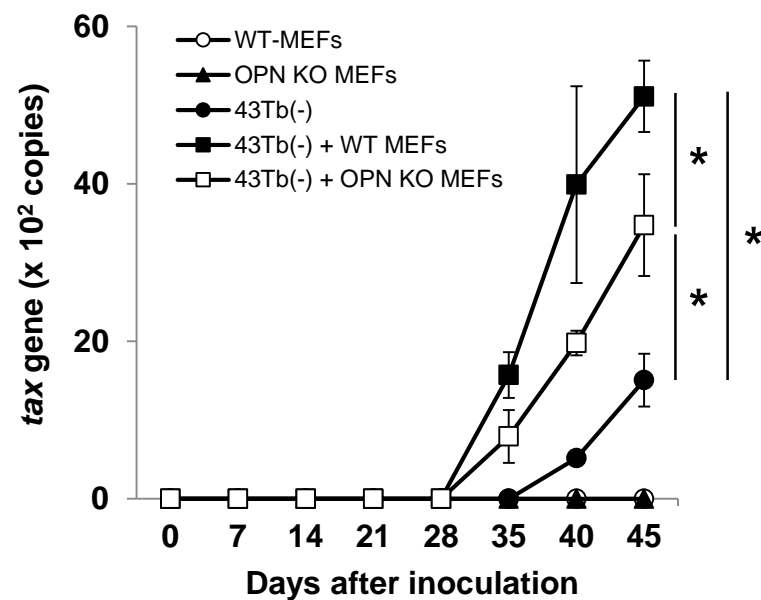

Supplement: Supplementary file 8 — 10.1186/s12977-015-0225-x OPN produced by MEFs promotes the tumor growth and metastasis of 43Tb(−) cells inoculated into NOG mice. Six-week-old female NOG mice were subcutaneously inoculated with 43Tb(−) cells alone (n = 3) or together with either WT MEFs (n = 3) or OPN KO MEFs (n = 3) (1 × 10^7^ cells per cell type). WT-MEFs (n = 1) or OPN KO MEFs (n = 1) only were also inoculated as negative controls. (A) The tumor size was measured every 3–4 days. (B) Blood was collected from the tail vein for detection of metastatic cells as quantified by the number of tax genes assessed using qPCR. For (A) and (B), bars indicate mean values ± SEM. Statistically significant differences are shown with P value (*P < 0.05, **P < 0.01). For (A) and (B), filled square, 43Tb(−) + WT MEFs; open square, 43Tb(−) + OPN KO MEFs; filled circle, 43Tb(−) only; open circle, WT MEFs; filled triangle, OPN KO MEFs. [file 12977_2015_225_MOESM8_ESM.pdf]
