# Supplementary material for: Osteopontin-integrin interaction as a novel molecular target for antibody-mediated immunotherapy in adult T-cell leukemia
Source: Retrovirology. 2015 Nov 24;12:99. doi: 10.1186/s12977-015-0225-x (PMC4657376; doi:10.1186/s12977-015-0225-x)
Supplement: Supplementary file 2 — 10.1186/s12977-015-0225-x Integrins and CD44 expression on T cell lines and primary cells associated with ATL. [file 12977_2015_225_MOESM2_ESM.docx]

**Supplementary Table S1 Integrins and CD44 expression on T cell lines and primary cells associated with ATL.**

**
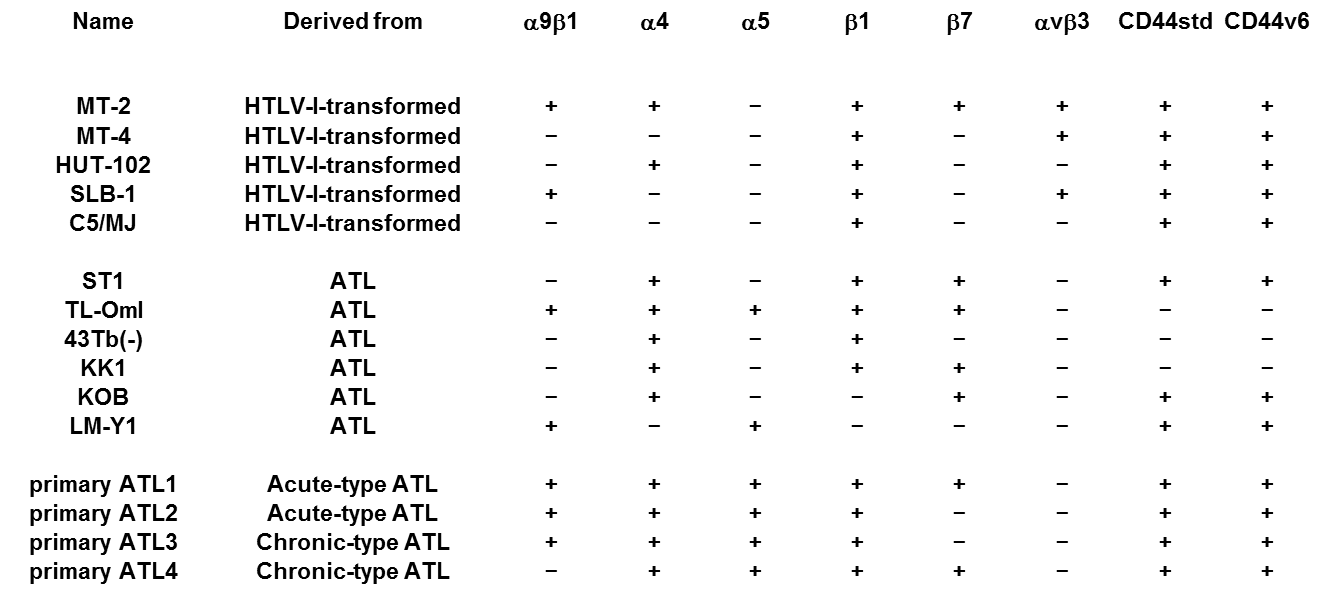
**

**Supplementary Table S1**

**Integrins and CD44 expression in cell lines and primary cells associated with ATL.**
